# Excellent 12-month outcomes after hip arthroscopy for femoroacetabular impingement: role of spinopelvic alignment and hip morphology—a multicenter study

**DOI:** 10.1186/s13018-026-06887-0

**Published:** 2026-05-07

**Authors:** Rémy Coulomb, Youssef Jamaleddine, Olivier May, Nicolas Bonin, Mathieu Thaunat, Nicolas Tardy, Nicolas Krantz, Pascal Kouyoumdjian

**Affiliations:** 1 Orthopedic and Traumatology Surgery Department, CHU Nîmes, Montpellier 1 University, Nîmes, France; 2 LMGC, Montpellier University, CNRS, Montpellier, France; 3 Department of Orthopedic Surgery, Lebanese American University Medical Center–Rizk Hospital, Beirut, Lebanon; 4 Médipôle Garonne, Clinique du Sport, 45 Rue de Gironis, 31036 Toulouse, France; 5 Lyon Ortho Clinic, 29B Avenue des Sources, 69009 Lyon, France; 6 Centre Orthopédique Santy, Ramsay Santé, Hôpital Privé Jean-Mermoz, 24 Avenue Paul-Santy, 69008 Lyon, France; 7 Centre ostéo-articulaire des Cèdres, Clinique des Cèdres, 5 Rue des Tropiques, 38130 Échirolles, France

**Keywords:** Femoroacetabular impingement, Hip arthroscopy, Spinopelvic alignment, Pelvic tilt, Outcome

## Abstract

**Background:**

Hip arthroscopy for femoroacetabular impingement yields favorable average results, yet many patients do not achieve truly excellent function. The influence of pre-operative radiographic and spinopelvic parameters on excellent short-term outcomes remains uncertain. We aimed to identify pre-operative radiographic and spinopelvic factors associated with achieving an excellent patient-reported outcome at 12 months after arthroscopic treatment of femoroacetabular impingement.

**Methods:**

A retrospective multicenter study of prospectively collected data was performed across six French centers. Consecutive patients treated arthroscopically for symptomatic femoroacetabular impingement between September 2020 and October 2021 were included if the 12-month Non-Arthritic Hip Score was available. Standard hip radiographs were used to measure degenerative grade, lateral centre-edge angle, and the Dunn alpha angle. Spinopelvic parameters were assessed on low-dose biplanar imaging in standing and sitting, including intrinsic pelvic mobility (standing minus sitting sacral slope). The primary endpoint was an excellent outcome defined as a 12-month Non-Arthritic Hip Score of at least 92. Missing spinopelvic data were handled by multiple imputation, and univariate then multivariable logistic regression was used, adjusted for age, sex, and body mass index.

**Results:**

Of 200 eligible patients, 178 (89.0%) had 12-month outcome data; 82 (46.1%) achieved a Non-Arthritic Hip Score of at least 92. In the adjusted model, standing pelvic tilt of 10 degrees or less (odds ratio 2.82; 95% confidence interval 1.35–5.90), degenerative grade 0 (odds ratio 5.15; 95% confidence interval 2.19–12.09), lateral centre-edge angle greater than 25 degrees (odds ratio 3.08; 95% confidence interval 1.41–6.74), and male sex (odds ratio 3.18; 95% confidence interval 1.38–7.32) independently predicted an excellent outcome. Intrinsic pelvic mobility, standing lumbar lordosis, Dunn alpha angle, age, and body mass index were not independently associated with excellent outcome.

**Conclusions:**

Only about half of patients undergoing hip arthroscopy for femoroacetabular impingement achieved an excellent 12-month outcome. Tonnis grade 0, lateral centre-edge angle greater than 25 degrees, standing pelvic tilt of 10 degrees or less, and male sex independently predicted excellent results, whereas pelvic mobility, standing lumbar lordosis, age, and body mass index did not.

## Background

Femoroacetabular impingement (FAI) is a dynamic conflict between the femoral head-neck junction and the acetabular rim caused by cam or pincer morphology, leading to labral and chondral damage, hip pain and loss of motion in young, active patients [1, 2]. Arthroscopic correction of cam or pincer deformities has become the preferred operative treatment for refractory FAI, with studies demonstrating superior pain relief and functional improvement compared with physiotherapy alone and favourable durability for many patients at long term follow-up [3, 4].

Nevertheless, a substantial proportion of patients do not achieve the level of symptom relief they consider fully satisfactory after hip arthroscopy. Studies applying the minimal clinically important difference (MCID) and patient acceptable symptomatic state (PASS) indicate that roughly one quarter of patients fail to reach clinically meaningful improvement [5, 6]. The Non-Arthritic Hip Score (NAHS) is a validated hip-specific patient-reported outcome measure for young individuals with non-arthritic hip pain and is widely used in FAI research [7]. Recent study has defined NAHS-specific MCID, PASS and substantial clinical benefit (SCB) thresholds in patients undergoing hip arthroscopy for FAI, with NAHS values around 92–94 corresponding to substantial clinical benefit, enabling the study of predictors of truly excellent outcomes rather than average score changes alone [8].

Nowadays, understanding of hip-spine interactions has advanced, initially in the context of total hip arthroplasty, where sagittal spinopelvic alignment and mobility influence functional cup position, impingement risk and instability [9, 10]. The concept of hip-spine syndrome has since been extended to young patients with non-arthritic hip disease, with low back pain shown to be common in FAI and associated with greater disability and worse hip-specific outcomes than in patients without back symptoms [11–13]. Recent FAI studies of static and dynamic spinopelvic parameters report conflicting results, with some suggesting that high pelvic incidence and abnormal sagittal alignment are associated with inferior outcomes after hip arthroscopy, whereas others have found little or no effect of spinopelvic morphology or lumbopelvic stiffness on short-term results [14–17], the studies have not evaluated these parameters in multivariable models, which may contribute to the conflicting findings.

However, most available work has evaluated radiographic and spinopelvic factors in relation to mean postoperative scores or generic thresholds such as the MCID or PASS, rather than to the achievement of an excellent level of function. The primary aim of the present multicentre observational study was therefore to identify pre-operative radiographic and spinopelvic parameters associated with achieving an excellent patient-reported outcome, defined as a NAHS of at least 92 at 12 months after arthroscopic treatment of FAI.

## Methods

### Study design and population

This is a retrospective multicenter study of prospectively collected data of patients who underwent hip arthroscopy for symptomatic FAI in six French medical centers. Eligible patients had clinical and radiographic evidence of cam or pincer morphology and an indication for arthroscopic treatment after failure of at least 6 months of structured non-operative management, including physiotherapy, sports adaptation or activity modification, and, sometimes, intra-articular injection. Patients with previous surgery on the index hip or concomitant procedures other than arthroscopic FAI correction were excluded. The study was approved by an Institutional Review Board at CHU-Nimes (IRB No. 26.01.05), and it was conducted in accordance with the principles of the Declaration of Helsinki. Participants received written information and were included according to a non-opposition procedure approved by the ethics committee.

For the present analysis, we included all consecutive patients from September 2020 to October 2021 with available NAHS at 12 months postoperatively. The primary endpoint was an excellent functional outcome at 12 months, defined as NAHS ≥ 92. Patients without 12-month NAHS data were excluded from outcome analyses.

### Clinical assessment and outcome measure

Pre-operative assessment included a standardized history and physical examination of the hip and lumbar spine. Patient-reported outcome measures comprised a hip pain visual analogue scale (0–10) and hip-specific questionnaires, including the NAHS [18]. The same instruments were administered 12 months after surgery. For this study, the primary functional outcome was the NAHS at 12 months, which was dichotomized as ≥ 92 (excellent outcome) versus < 92 (non-excellent outcome).

### Radiographic and spinopelvic assessment

All patients underwent a standard radiographic work-up including a standing anteroposterior pelvic radiograph, a lateral hip view, and a 45° Dunn view. From these images, the lateral centre-edge (LCE) angle and the Dunn alpha angle were measured, and degenerative changes were graded using the Tönnis classification. Advanced imaging (magnetic resonance imaging or arthro-computed tomography) was obtained when clinically indicated but was not used as a source of candidate predictors.

**Fig. 1:**
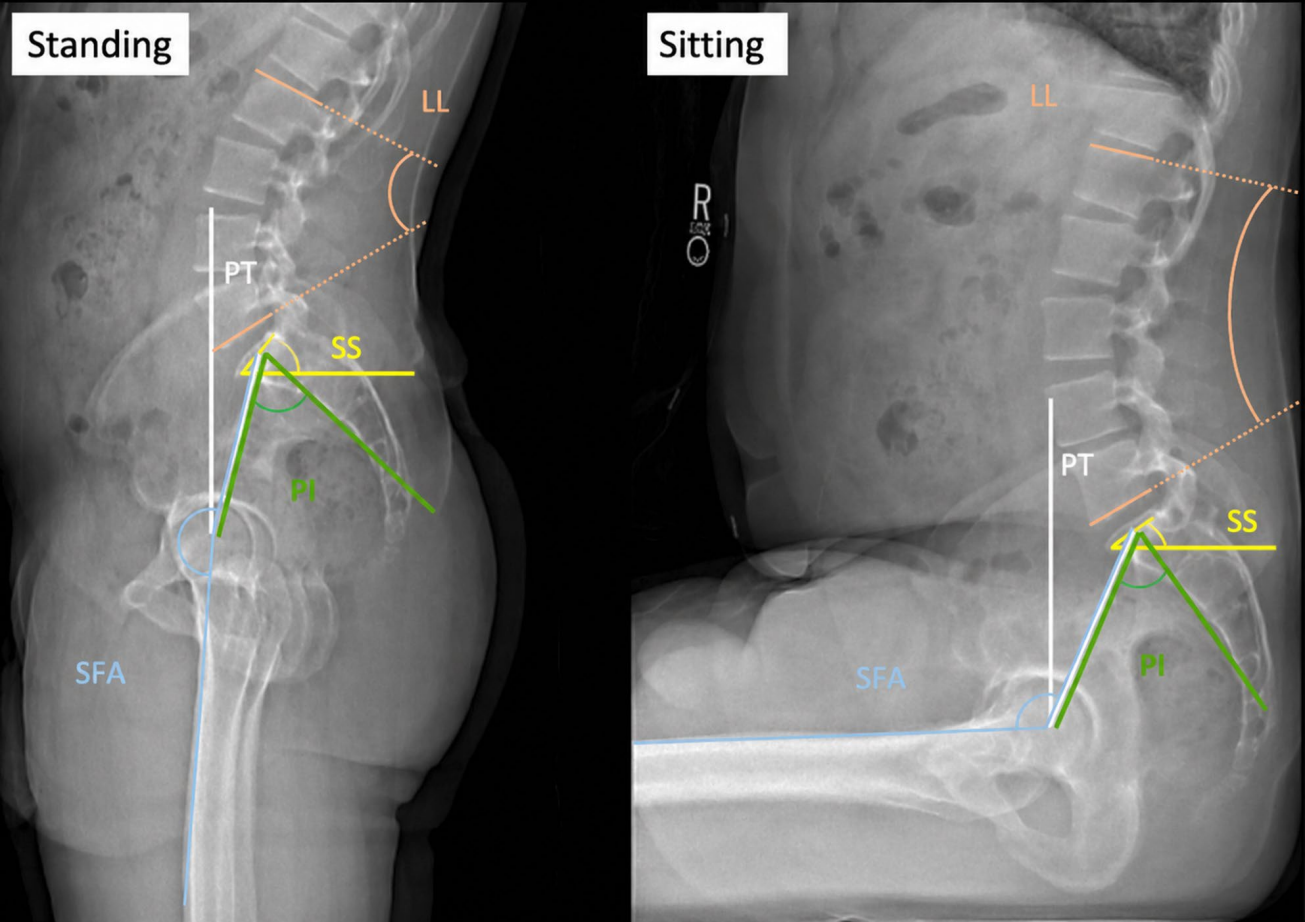
Spinopelvic parameters measured on standing and sitting lateral EOS images. Lumbar lordosis (LL), pelvic tilt (PT), sacral slope (SS), pelvic incidence (PI), and sacro-femoral angle (SFA). Intrinsic pelvic mobility (IPM) was defined as the difference in sacral slope between standing and sitting positions

Spinopelvic parameters were assessed using a low-dose biplanar EOS imaging system (EOS imaging, SA 10 rue Mercoeur 75011 Paris, France) in standing and sitting positions. Recorded sagittal parameters included pelvic incidence, sacral slope, pelvic tilt, lumbar lordosis, sacro-femoral angle, and femoral version. The main sagittal spinopelvic parameters assessed on standing and sitting lateral EOS images are illustrated in Fig. 1. Intrinsic pelvic mobility (IPM) was defined as the difference in sacral slope between standing and sitting (IPM = sacral slope standing - sacral slope sitting).

### Arthroscopic surgical technique

Arthroscopic treatment was performed according to each surgeon’s standard technique in the participating centers. Briefly, procedures were performed with the patient in the supine position under traction through standard anterolateral and mid-anterior portals. Depending on surgeon preference, access was obtained either through a central-compartment-first or a peripheral-compartment-first approach. Diagnostic arthroscopy was followed by treatment of labral and chondrolabral pathology as indicated, femoral osteochondroplasty for cam correction, and acetabular rim trimming for pincer morphology when required. Capsular management, including closure or plication, was performed according to intraoperative findings and surgeon preference; however, in some centers, the capsule was routinely closed, particularly in patients with a lateral centre-edge angle of less than 25°.

### Candidate predictors

Pre-operative radiographic and spinopelvic variables were selected a priori as candidate predictors. The primary spinopelvic predictor included in the models was IPM which was dichotomized using a threshold of > 10°. Standing pelvic tilt was dichotomized at ≤ 10°, reflecting a more anteriorly rotated pelvis in standing. Tönnis grade was dichotomized as 0 versus ≥ 1 to distinguish non-degenerative from early degenerative changes. The LCE angle was dichotomized at > 25° to indicate adequate acetabular coverage. Standing lumbar lordosis and the Dunn alpha angle were analysed as continuous variables (per degree).

In addition, age at index surgery, sex, and body mass index were considered demographic confounders and were included in multivariable models. These variables were recorded prospectively at baseline.

### Data analysis

There was some missing data involving EOS-derived spinopelvic parameters. In contrast, standard pre-operative radiographic variables on the index hip (Tönnis grade, lateral centre-edge angle, Dunn alpha angle), baseline NAHS, and clinical covariates (age, sex, and BMI) were complete with no missing values. To minimize bias and avoid excluding a substantial number of patients, missing values were handled using multiple imputation by fully conditional specification (chained equations), with 20 imputed datasets generated under a missing-at-random assumption. The imputation model included the 12-month NAHS outcome, all candidate predictors, and relevant auxiliary radiographic and clinical variables. Patients with missing primary outcome (12-month NAHS) were excluded from the study and analysis. Categorical variables are described as frequencies and percentages, and continuous variables as medians with interquartile ranges. The primary endpoint was an excellent functional result, defined as NAHS ≥ 92 at 12 months. Univariate logistic regression models were first fitted in each dataset to explore the association between each candidate predictor and achievement of NAHS ≥ 92, followed by a multivariable logistic regression model including all pre-operative radiographic and spinopelvic variables with adjustment for age, sex and BMI. For both univariate and multivariable analyses, regression coefficients and standard errors from each dataset were combined using Rubin’s rules to obtain pooled odds ratios (ORs) with 95% confidence intervals (CI). All tests were two-sided, and a p-value < 0.05 was considered statistically significant. All missing data handling and statistical analyses were performed using IBM SPSS Statistics for Windows, Version 27.0 (IBM Corp., Armonk, NY, USA).

## Results

A total of 200 patients were included initially. Of these, 178 patients (89.0%) had available 12-month NAHS data and were analysed, and 22 patients (11.0%) without 12-month NAHS were excluded from outcome analyses. At 12 months, 82 patients (46.1%) had an excellent outcome (NAHS ≥ 92), and 96 patients (53.9%) had NAHS < 92.

Patient characteristics are summarized in Table 1.

**Table 1:** Baseline patient characteristics by 12-month NAHS status

Characteristic	Overall (*n* = 178)	NAHS < 92 (*n* = 96)	NAHS ≥ 92 (*n* = 82)	*p*-value
Age (years)	28.7 [23.5–36.6]	30.3 [23.9–37.4]	27.8 [23.1–35.9]	0.210
Male sex	119 (66.9%)	59 (61.5%)	60 (73.2%)	0.112
BMI (kg/m²)	23.2 [21.4–24.8]	23.4 [21.3–24.9]	23.0 [21.6–24.8]	0.944

Distributions of pre-operative radiographic and spinopelvic parameters according to 12-month NAHS status are summarised in Tables 2 and 3. Values are based on available (non-imputed) data, and denominators vary for EOS-derived measures due to missing measurements.

**Table 2:** Categorical preoperative radiographic and spinopelvic parameters by 12-month NAHS status

Pre-operative radiographic and spinopelvic parameters		NAHS < 92 (*n*, %)	NAHS ≥ 92 (*n*, %)	Total (*n*)	*p*-value
Intrinsic pelvic mobility > 10°	Yes	65 (77.4%)	61 (83.6%)	126	0.442
	No	19 (22.6%)	12 (16.4%)	31	
Standing pelvic tilt ≤ 10°	Yes	35 (36.5%)	46 (56.1%)	81	0.013
	No	61 (63.5%)	36 (43.9%)	97	
Tönnis grade 0	Yes	53 (55.2%)	69 (84.1%)	122	< 0.001
	No	43 (44.8%)	13 (15.9%)	56	
Lateral centre-edge angle > 25°	Yes	60 (62.5%)	68 (82.9%)	128	0.004
	No	36 (37.5%)	14 (17.1%)	50	

**Table 3:** Continuous pre-operative radiographic parameters by 12-month NAHS status

Continuous radiographic parameters	NAHS < 92		NAHS ≥ 92		*p*-value
	(*n*)	median [Q1–Q3]	(*n*)	median [Q1–Q3]	
Standing lumbar lordosis (°)	80	45.0 [37.0–55.0]	60	55.0 [42.0–61.0]	0.014
Preoperative Dunn alpha angle (°)	96	68.0 [60.0–74.0]	82	70.0 [55.8–75.8]	0.639

In univariate logistic regression analyses after multiple imputation, standing pelvic tilt ≤ 10°, Tönnis grade 0, and lateral centre-edge angle > 25° were each associated with higher odds of achieving NAHS ≥ 92 at 12 months. Standing pelvic tilt ≤ 10° was associated with an unadjusted odds ratio (OR) of 2.23 (95% confidence interval [CI] 1.22–4.07; *p* = 0.009). Tönnis grade 0 (vs. ≥ 1) showed an unadjusted OR of 4.31 (95% CI 2.10–8.81; *p* < 0.001), and lateral centre-edge angle > 25° an unadjusted OR of 2.91 (95% CI 1.44–5.92; *p* = 0.003). Intrinsic pelvic mobility > 10° (OR 1.44; 95% CI 0.65–3.18; *p* = 0.367), standing lumbar lordosis (per 1°; OR 1.02; 95% CI 1.00–1.04; *p* = 0.069), pre-operative Dunn alpha angle (per 1°; OR 1.01; 95% CI 0.98–1.03; *p* = 0.584), age, BMI, and male sex were not significantly associated with achieving NAHS ≥ 92 in univariate analyses.

In the multivariable logistic regression model including all pre-operative radiographic and spinopelvic variables, with age, sex, and BMI entered as covariates, three pre-operative parameters remained independently associated with achieving an excellent outcome (NAHS ≥ 92). Standing pelvic tilt ≤ 10° was associated with higher odds of NAHS ≥ 92 (adjusted OR 2.82; 95% CI 1.35–5.90; *p* = 0.006). Tönnis grade 0 (vs. ≥ 1) showed the strongest association (adjusted OR 5.15; 95% CI 2.19–12.09; *p* < 0.001). Lateral centre-edge angle > 25° also remained independently associated with NAHS ≥ 92 (adjusted OR 3.08; 95% CI 1.41–6.74; *p* = 0.005). Intrinsic pelvic mobility > 10°, standing lumbar lordosis, pre-operative Dunn alpha angle, age at inclusion, and BMI were not independently associated with NAHS ≥ 92 in the adjusted model. Male sex (vs. female) emerged as an independent predictor (adjusted OR 3.18; 95% CI 1.38–7.32; *p* = 0.007).

Univariate and multivariable logistic regression results for predictors of NAHS ≥ 92 at 12 months are summarised in Table 4.

**Table 4:** Univariate and multivariable logistic regression for predictors of NAHS ≥ 92 at 12 months

Predictor	Unadjusted OR (95% CI)	*p*-value	Adjusted OR (95% CI)	*p*-value
Intrinsic pelvic mobility > 10°	1.44 [0.65; 3.18]	0.367	1.05 [0.43; 2.52]	0.921
Standing pelvic tilt ≤ 10°	2.23 [1.22; 4.07]	**0.009**	2.82 [1.35; 5.90]	**0.006**
Tönnis grade 0 (vs. ≥ 1)	4.31 [2.10; 8.81]	< 0.001	5.15 [2.19; 12.09]	**< 0.001**
Lateral centre-edge angle > 25°	2.91 [1.44; 5.92]	**0.003**	3.08 [1.41; 6.74]	**0.005**
Standing lumbar lordosis (per 1°)	1.02 [1.00; 1.04]	0.069	1.01 [0.98; 1.03]	0.649
Preoperative Dunn alpha angle (per 1°)	1.01 [0.98; 1.03]	0.584	0.99 [0.95; 1.02]	0.381
Age (per 1 year)	0.97 [0.94; 1.01]	0.110	0.99 [0.95; 1.03]	0.685
Body mass index (per 1 kg/m²)	1.00 [0.90; 1.10]	0.950	1.09 [0.96; 1.23]	0.203
Male sex (vs. female)	1.71 [0.90; 3.24]	0.099	3.18 [1.38; 7.32]	**0.007**

Statistically significant* p*-values are shown in bold (*p* < 0.05).

## Discussion

Our study shows that only about half of patients undergoing hip arthroscopy for FAI achieved an “excellent” postoperative result (NAHS ≥ 92), despite overall good improvement in symptoms. In that context, achieving merely “acceptable” outcomes (MCID or PASS) is common [19], but reaching a truly high-functioning, near-asymptomatic state may be more demanding.

The strong positive association between Tönnis grade 0 and excellent outcome underscores the pivotal role of preservation of joint cartilage. Patients without radiographic signs of osteoarthritis had markedly higher odds of reaching NAHS ≥ 92, whereas degenerative changes were detrimental. This aligns with long-term data showing that patients with Tönnis < 1 have > 90% survivorship and superior PROMs after hip arthroscopy, while chondral damage and older age are the most consistent predictors of conversion to THA [4]. These findings reinforce the concept of FAI as a pre-arthritic condition in which early intervention before structural joint deterioration offers the greatest potential for durable benefit [1, 2].

Similarly, greater lateral center-edge angle (> 25°) independently predicted excellent outcome, suggesting that global acetabular coverage remains an important condition for successful arthroscopic correction of FAI. Patients with lower LCE values were less likely to attain NAHS ≥ 92 despite correction of cam and pincer morphology. However, another study found that patient who had hip arthroscopy with capsular plication for FAI showed substantial clinical improvement and needed few additional surgeries, whether their acetabulum showed borderline dysplasia or normal coverage [20]. In addition, another study found that after arthroscopic FAI surgery with capsular closure, clinical success rates were similar in patients with borderline hip dysplasia (BHD) and those with normal coverage, but in BHD patients, a larger preoperative LCEA predicted a higher chance of success [21]. Taken together, these data support the interpretation that borderline undercoverage does not preclude clinically meaningful benefit from arthroscopy, but greater acetabular coverage may favor the achievement of excellent outcomes (NAHS ≥ 92).

An important finding of this study is the role of static sagittal alignment. A more anteriorly rotated pelvis in standing (pelvic tilt ≤ 10°) almost tripled the odds of achieving excellent outcomes (NAHS ≥ 92). This complements a recent study, which found that high pelvic incidence (> 60°), a spinopelvic parameter typically associated with more posterior functional pelvic tilt, predicted inferior PROMs and lower rates of achieving clinically meaningful thresholds after hip arthroscopy for FAI and labral tears [15]. These observations support the hip-spine concept, in which the lumbo-pelvi-femoral complex behaves as a single functional unit, a concept described in total hip arthroplasty [22].

In contrast, intrinsic pelvic mobility (IPM > 10° versus ≤ 10°) and lumbar lordosis were not associated with attainment of excellent outcome in our multivariable model. This is consistent with a study that reported that FAI patients more often have a stiff spine than controls, but found no difference in 1-year postoperative PROMs between stiff and non-stiff groups [14]. Similarly, a study showed that limited lumbopelvic mobility did not adversely affect short-term outcomes or rates of MCID or PASS after primary hip arthroscopy [17]. From a biomechanical perspective, spinopelvic mobility remains relevant in FAI, as posterior pelvic tilt has been shown to increase impingement-free hip range of motion, highlighting the close relationship between pelvic motion, femoroacetabular conflict, and functional hip ROM [23]. Our results therefore support a growing body of evidence that static spinopelvic morphology may be more important than sagittal mobility per se in determining hip specific outcomes after arthroscopy.

Finally, male sex was an independent predictor of excellent outcome in our cohort. Prior work has shown that adolescents and young adults of both sexes can achieve high rates of MCID and PASS after hip arthroscopy for FAI [6]. However, a systematic review showing heterogeneous sex-based results after hip arthroscopy, with 29% of the studies reported female sex as a negative predictor of postoperative outcomes, 13% as a positive predictor, and 58% reported no sex-based difference [24]. Our data suggest that, even in a relatively young, nonarthritic population, female patients may be less likely to reach the excellent outcomes (NAHS ≥ 92). This should inform preoperative counselling and reinforces the value of reporting absolute thresholds (such as NAHS ≥ 92) in addition to change scores in the studies.

This study has several limitations. First, it was a retrospective analysis of prospectively collected multicenter data and is therefore subject to selection bias, center-related heterogeneity, and residual confounding despite multivariable adjustment. Second, 22 of 200 eligible patients did not have 12-month NAHS data and were excluded from the outcome analysis, which may have introduced attrition bias. Third, some EOS-derived spinopelvic variables were missing and required multiple imputation. In addition, hip ROM was not available as a standardized multicenter variable and therefore could not be directly correlated with spinopelvic parameters or postoperative outcome. Fourth, spine pathology was not comprehensively characterized beyond EOS-derived alignment parameters, and concomitant symptomatic lumbar disease may have influenced both spinopelvic mechanics and postoperative outcomes. Nevertheless, by combining standard hip radiographs with standing EOS measures and anchoring our endpoint to validated NAHS thresholds, we propose practical imaging-based criteria to help identify patients most likely to achieve truly excellent early outcomes after hip arthroscopy.

## Conclusion

This multicentre study shows that only about half of patients undergoing hip arthroscopy for FAI achieved an excellent 12-month outcome (NAHS ≥ 92). Tönnis grade 0, lateral centre-edge angle > 25°, standing pelvic tilt (≤ 10°), and male sex were independent predictors of excellent results, whereas intrinsic pelvic mobility, standing lumbar lordosis, age and BMI were not predictive. These imaging-based and demographic criteria may help in patient counselling, and should be validated in larger cohorts with longer follow-up.

## Data Availability

The datasets generated and analysed during the current study are not publicly available due to patient privacy.

## Abbreviations

AP: Anteroposterior
BHD: Borderline hip dysplasia
BMI: Body mass index
CI: Confidence interval
CT: Computed tomography
EOS: EOS biplanar low-dose imaging system
FAI: Femoroacetabular impingement
IPM: Intrinsic pelvic mobility
IQR: Interquartile range
LCE (angle): Lateral centre-edge (angle)
LCEA: Lateral centre-edge angle
MCID: Minimal clinically important difference
MRI: Magnetic resonance imaging
NAHS: Non-Arthritic Hip Score
OR: Odds ratio
PASS: Patient acceptable symptomatic state
PROM(s): Patient-reported outcome measure(s)
Q1–Q3: First to third quartile
SCB: Substantial clinical benefit
SPSS: Statistical Package for the Social Sciences
THA: Total hip arthroplasty
